# Visual saliency models for summarization of diagnostic hysteroscopy videos in healthcare systems

**DOI:** 10.1186/s40064-016-3171-8

**Published:** 2016-09-06

**Authors:** Khan Muhammad, Jamil Ahmad, Muhammad Sajjad, Sung Wook Baik

**Affiliations:** 1 Intelligent Media Laboratory, Department of Digital Contents, College of Electronics and Information Engineering, Sejong University, Seoul, Republic of Korea; 2 Digital Image Processing Laboratory, Department of Computer Science, Islamia College Peshawar, Khyber Pakhtunkhwa, Pakistan

**Keywords:** Video summarization, Image and video processing, Medical image analysis, Visual saliency models, Diagnostic hysteroscopy

## Abstract

In clinical practice, diagnostic hysteroscopy (DH) videos are recorded in full which are stored in long-term video libraries for later inspection of previous diagnosis, research and training, and as an evidence for patients’ complaints. However, a limited number of frames are required for actual diagnosis, which can be extracted using video summarization (VS). Unfortunately, the general-purpose VS methods are not much effective for DH videos due to their significant level of similarity in terms of color and texture, unedited contents, and lack of shot boundaries. Therefore, in this paper, we investigate visual saliency models for effective abstraction of DH videos by extracting the diagnostically important frames. The objective of this study is to analyze the performance of various visual saliency models with consideration of domain knowledge and nominate the best saliency model for DH video summarization in healthcare systems. Our experimental results indicate that a hybrid saliency model, comprising of motion, contrast, texture, and curvature saliency, is the more suitable saliency model for summarization of DH videos in terms of extracted keyframes and accuracy.

## Background

The recent advancement in modern technology has shown promising results in human reproductive healthcare. One of the popular method of ensuring reproductive health is diagnostic hysteroscopy (DH), where the sensitive regions of female reproductive system (FRS) are assessed and visualized to diagnose uterine abnormalities (Gavião et al. 2012). An application of the DH is evaluation of glandular openings, concerning with prognosis and reproductive status and other abnormities mentioned in Gavião and Scharcanski (2007). The DH procedure is performed by a gynecologist using a hysteroscope, which can disseminate the captured sequence of frames to a screen. The DH is performed several times per day, producing a number of DH videos, each of average length 3–4 min. These videos are fully recorded by hospitals in long-term video libraries for later inspection of previous diagnosis, research and training, and as an evidence for patients’ complaints in courts (Scharcanski et al. 2006). However, from diagnosis point of view, a small number of frames are required for gynecologists to diagnose the abnormality. To this end, gynecologists mostly browse the recorded DH videos manually to select the representative frames for supporting DH and as a record in patient history, making this process tedious and time consuming compared to the actual DH examination (Gavião and Scharcanski 2007).

**Fig. 1:**
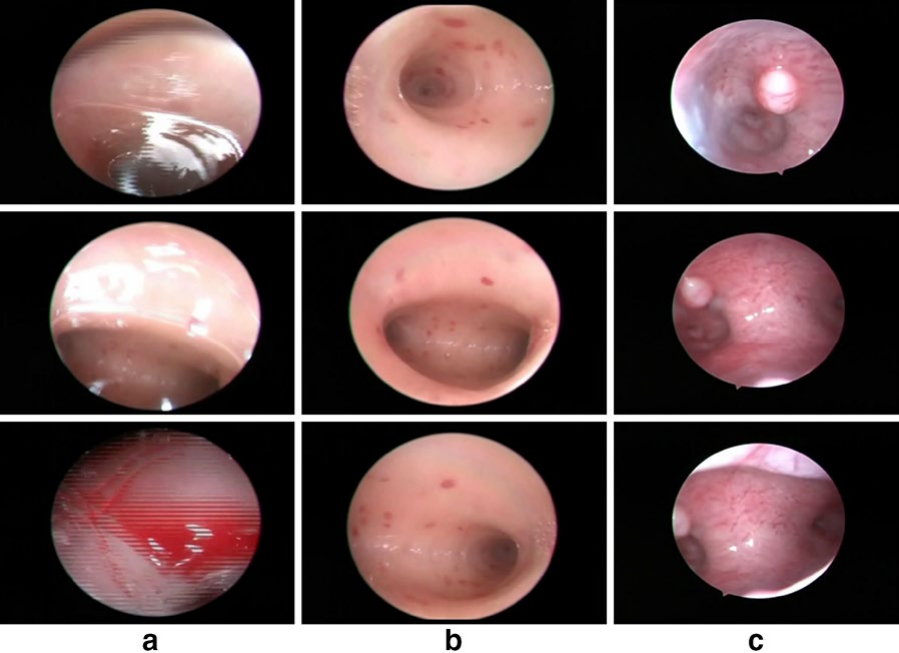
**a** Non-important frames, indicating irrelevant DH frames contaminated by lighting and biological effects, **b**, **c** important frames representing diagnostically important DH frames from relevant DH video segments

To avoid this time consuming task, video prioritization schemes can be explored to extract keyframes, allowing gynecologists for non-linear browsing of DH contents. Consequently, the extracted keyframes can be used for efficient indexing of DH videos and generation of video summaries, containing relevant DH contents (Ejaz et al. 2013). To evaluate such VS schemes, gynecologists are requested to suggest portions of DH videos, which are diagnostically important and can be represented by a frame. From diagnostic point of view, the video portions with unobstructed view of FRS are important for gynecologists as illustrated in Fig. 1b, c. The DH frames contaminated by lighting and biological effects are discarded and are not of interest to gynecologists. An example of such irrelevant frames is shown in Fig. 1a.

During the DH examination, the specialist spends most of his time in searching for clinically important regions of FRS. Once such areas are found, the hysteroscope is focused on the areas of interest to capture numerous frames (Gavião and Scharcanski 2005). In addition, the surrounding areas near the region of interest are also examined by slowly moving the hysteroscope. Thus, the DH videos contain an enormous amount of redundant frames due to more examination of region of interest and low camera motion. Conversely, the non-important regions are examined quickly with fast movement of hysteroscope (Gavião et al. 2012).

Considering the aforementioned concerns, in this paper, we evaluate the performance of different general-purpose and domain specific VS methods for extraction of keyframes from DH videos. The study covers motion (Mehmood et al. 2015), texture (Ejaz et al. 2013), multi-scale contrast (Mehmood et al. 2014), curvature (Mehmood et al. 2013), and saliency detection using information maximization (SIM) (Bruce and Tsotsos 2005) for summarization. In addition, two general-purpose VS techniques (Ejaz et al. 2012; Ejaz and Baik 2012) are also considered for comparative analysis. The selection of best saliency detection model for summarization of DH videos is then suggested based on an evaluation criteria, reflecting computational complexity and accuracy.

The rest of this paper is organized as follows: “Related work” section presents an overview of video summarization and its related schemes. The details of this study are illustrated in “Methods” section. “Experimental results and discussion” section presents the experimental results, followed by concluding remarks and future directions in “Conclusion” section.

## Related work

In this section, we present an overview of video summarization along with the previous works related to the process of DH videos abstraction. VS refers to identification of pertinent contents in a video for producing its concise representation known as video abstracts, which can be of two types (Truong and Venkatesh 2007): keyframes extraction and video skims. The former type is concerned with extraction of salient frames from the video. The latter category of VS extracts a condensed form of video clip with short duration, highlighting the main contents of original video (Ejaz et al. 2013). To produce a video abstract, there are two possible ways including manual and automatic summarization. Due to an enormous volume of video data, manual keyframes extraction is difficult and time consuming. Therefore, it is necessary to explore automatic VS for efficient utilization of manpower and other resources.

The current literature indicates that two major categories of features have been used for summarization including low and high-level features. Low-level features based VS methods (Ejaz et al. 2012; De Avila et al. 2011; Almeida et al. 2012; De Avila et al. 2008; Almeida et al. 2013) utilize numerous low-level features such as moments, color, motion, and shape. Due to semantic gap, the low-level features based VS methods do not agree with high-level human perception, decreasing its applicability. Considering this problem, researchers incorporated visual attention models in summarization methods, which extract frames reflecting the human attention. The first visual-attention directed VS scheme is proposed by Ma et al. (2005), utilizing visual, linguistic, and aural features for summary generation. Ejaz et al. (2013) presented a general-purpose keyframes extraction approach utilizing visual attention model. The method is utilizing temporal-gradient directed dynamic visual saliency, which is computationally inexpensive compared to traditional optical flow approaches. In addition, the static visual saliency based on DCT^1^ is incorporated in the proposed framework. A non-linear weighted fusion is then used to combine the static and dynamic visual attention measures for generating an attention curve, which is used for producing a video summary.

The previous literature shows that visual attention model based VS schemes are most efficient in finding semantically relevant video summaries in contrast to low-level features based VS methods (Mehmood et al. 2016). Therefore, the focus here is to explore visual attention models based VS methods for extraction of diagnostically important frames from DH videos.

Scharcanski et al. (2006) presented a VS scheme for extraction of clinically important segments, facilitating quick browsing of DH videos for desired contents. Their scheme can be used to extract keyframes, which are used in record management of patients. Their presented scheme consists of two main steps: (1) firstly, a set of significant video segments are selected using statistical methods and (2) secondly, a post-processing step combines the similar adjacent video segments, avoiding over-segmentation. Gavião and Scharcanski (2007) nominated a VS method for detection of clinically significant segments in DH videos and extracting frames, providing a better visualization of the endometrium details such as glandular openings and vascularization. The approach can generate a video summary containing pertinent frames, enabling quick browsing of video contents. The proposed technique utilizes singular value decomposition characteristics during video abstraction, avoiding parameter adjustment.

Gavião et al. (2012) introduced another method for extraction of clinically important segments for DH videos. The method is capable of associating clinical significance with a DH video clip during the examination session of DH by gynecologist. Using the results of this method, the gynecologists can browse a given DH video non-linearly, saving their analysis time in manually visualizing each frame of the video. Another recent VS method for DH video abstraction is presented by Ejaz et al. (2013), where multi-scale contrast, motion, and texture based saliencies are combined for making a visual attention curve. The keyframes are then extracted using this attention curve, which can be used for analysis and indexing of DH videos.

The above literature designates that numerous proposals have been presented for general-purpose video summarization and DH video abstraction, considering individual factors such as efficiency, computational complexity, and accuracy. The previous VS methods are either too naïve or too complex with significant computational cost. The complex schemes achieve better accuracy in terms of keyframe extraction, however, their extensive computational cost make them less suitable for real-time summarization such as keyframes extraction during wireless capsule endoscopy (Mehmood et al. 2014; Muhammad et al. 2016). The VS methods utilizing simple features are computationally cost-effective, however, their lower accuracy makes them infeasible for sensitive areas of interest such as DH video summarization (Ejaz et al. 2013) and orthoscopic video summarization (Lux et al. 2010). It is therefore important to explore the general-purpose and domain-specific VS methods and exploit a VS framework for keyframes extraction from DH videos, which can maintain a balance between computation cost and accuracy.

## Methods

In this section, we describe the mechanism of all the VS methods, which are considered for evaluation in terms of keyframes extraction and accuracy for DH videos. The methods under consideration include two general-purpose VS schemes, a general-purpose saliency detection model, and numerous domain-specific visual saliency detection models for medical videos. The general-purpose VS methods are our previous works including low-level features based VS (Ejaz et al. 2012) and high-level features based VS (Ejaz et al. 2013). In the former work, three low-level features such as correlation, histogram, and moments of inertia are extracted from the underlying video, which are fused using an aggregation mechanism. An adaptive mechanism is utilized during summarizing the video by combining the intermediate results, reducing the redundancy. Finally, the keyframes are extracted based on the attention values obtained using the aggregation mechanism.

In the second general-purpose VS method (Ejaz et al. 2013), keyframes are extracted using high-level features of visual attention model. The main bedrocks of this approach is incorporation of temporal-gradient directed dynamic visual saliency and DCT based static visual saliency for summarization, which are computational inexpensive compared to traditional optical-flow schemes. A non-linear weighted fusion is then used to combine the static and dynamic visual attention measures for generating an attention curve, which is used for keyframes extraction. In the coming sub-sections, we describe the various saliency detection models particularly used in summarization of DH videos.

### Motion saliency

Motion saliency is one of the prominent saliency detection models used for video summarization in general (Mehmood et al. 2015) and DH video abstraction in particular (Ejaz et al. 2013). In the context of DH videos, motion saliency is effective in finding the inter-frame motion, providing a clue about the importance of a frame. During the DH examination, the gynecologist spends little time in examining the non-important areas by quickly moving the hysteroscope, producing fast inter-frame motion. On the other hand, more time is spend in visualizing the areas of interest by slowly moving the hysteroscope (Ejaz et al. 2013). This produces a significant amount of redundant frames with low inter-frame motion. This gives a clue that the keyframes lie in the sequence of frames having less inter-frame motion. The motion saliency is computed using Eq. 1 as follows:

1 $$M\left( {DHF_{i} ,P} \right) = \sqrt {M_{x}^{2} \left( P \right) + M_{y}^{2} \left( P \right)}$$

Herein, $M_{x} \left( P \right)$ and $M_{y} \left( P \right)$ indicate the x and y components of the motion vector at pixel “P” of the DH frame “DHF_i_” relative to the previous frame “DHF_i-1_”. After computing the motion saliency for each frame, the obtained saliency values are normalized in the range of 0–1.

### Texture saliency

In the domain of DH video abstraction, texture saliency can be used to identify the most injurious areas of DH frames. For this purpose, an entropy-directed texture segmentation approach is used. The texture saliency for a DH frame “DHF” can be calculated as follows:

2 $$E\left( {DHF,P} \right) = - \sum\limits_{k = 0}^{\eta - 1} {Hist_{P} \left( k \right)} \log_{2} \left( {Hist_{P} \left( k \right)} \right)$$

3 $$TXI\left( {DHF,P} \right) = \left\{ {\begin{array}{*{20}l} 0 \hfill &\quad {if\,E\left( {DHF,P} \right) < \tau } \hfill \\ 1 \hfill & \quad{otherwise} \hfill \\ \end{array} } \right.$$

4 $$TS\left( {DHF,P} \right) = \left\{ {\begin{array}{*{20}l} {DHF\left( P \right)} \hfill & {if\,TXI\left( {DHF,P} \right) = 1} \hfill \\ 0 \hfill & {if\,TXI\left( {DHF,P} \right) = 0} \hfill \\ \end{array} } \right.$$

Firstly, the entropy “E” of pixel “P” at frame “DHF” is calculated using Eq. 2. A texture segmentation with τ = 0.8 is then applied on “E” as shown in Eq. 3, resulting an injury-free texture image “TXI”. Then the edges of “TXI” are smoothened using closing. Next, the holes in “TXI” are filled, providing the mask image, based on which the injurious parts of the DH frame can be identified (Ejaz et al. 2013). It is worth mentioning that texture saliency “TS” contains only the injurious regions of DH frame. Therefore, a salient frame in this context is the one, whose larger area is injurious. Alternatively, the DH frame with high proportional of injurious regions is assigned a saliency value of 1. The remaining of the frames get their saliency scores relative to the maximum value. To sum up, texture saliency effectively segments the injurious parts of DH frames and assigns them higher saliency scores compared to frames with low-proportional of injurious regions.

### Multi-scale contrast map

Contrast map is an effective measurement for finding the uniqueness of a region in a video frame, which has been widely used in computer vision algorithms (Ejaz et al. 2013; Perazzi et al. 2012). In the context of DH video summarization, we explore multi-scale color contrast, which is more effective in salient objects identification of different sizes. The multi-scale color contrast map of a DH frame is calculated in Eqs. 5 and  6 as follows:

5 $$CCM_{c}^{l} \left( {DHF_{c} ,P} \right) = \sum\limits_{q \in N\left( P \right)} {\left\| {DHF_{c}^{l} \left( P \right) - DHF_{c}^{l} \left( q \right)} \right\|}^{2}$$

6 $$MSCCM\left( {DHF,P} \right) = \sum\limits_{l = 1}^{\eta } {CCM^{l} \left( {DHF,P} \right)}$$

Herein, “DHF_c_” indicates one of the three color channels (red, green or blue) for the frame “DHF”. “*l*” refers to the scale of contrast and “N(p)” shows the neighborhood of the pixel “P”, which is 5 × 5. The value of ɳ is set to 3, indicating the levels of Gaussian pyramid (Liu et al. 2011).

### Curvature map

During DH examination, the gynecologists move the hysteroscope with a certain orientation to effectively visualize the areas of interests. The previously mentioned saliency detection models are less effective in handling DH frames with such abnormalities. In this context, curvature map is comparatively more effective due to its rotational-invariant property in finding the keyframes with abnormalities, which are captured from different orientations. Furthermore, the neuroscience and psychophysical research also dictates that curvature is an important factor in determining the saliency and improving the decision of gynecologists in selection of keyframes. The curvature map “CM” for a DH frame “DHF” can be calculated using Eqs. 7 and 8 as follows (Mehmood et al. 2013):

7 $$CM = \left| {\nabla^{2} g} \right| = \sqrt {g_{xy}^{2} + g_{xx}^{2} + g_{yx}^{2} + g_{yy}^{2} }$$

8 $$\left\{ {\begin{array}{*{20}l} {g\,\left( {x,y} \right) = DHF\left( {x,y} \right) \times \varPsi } \hfill \\ {\varPsi = e^{{ - \,\,\frac{{x^{2} + y^{2} }}{{2\sigma^{2} }}}} } \hfill \\ {g_{xy} = \frac{{\partial^{2} g}}{{\partial_{x} \partial_{y} }},\quad g_{xx} = \frac{{\partial^{2} g}}{{\partial_{{x^{2} }} }},\quad g_{yx} = \frac{{\partial^{2} g}}{{\partial_{y} \partial_{x} }},\quad g_{yy} = \frac{{\partial^{2} g}}{{\partial_{{y^{2} }} }}} \hfill \\ \end{array} } \right\}$$

### Fusion scheme and extraction of keyframes

**Fig. 2:**
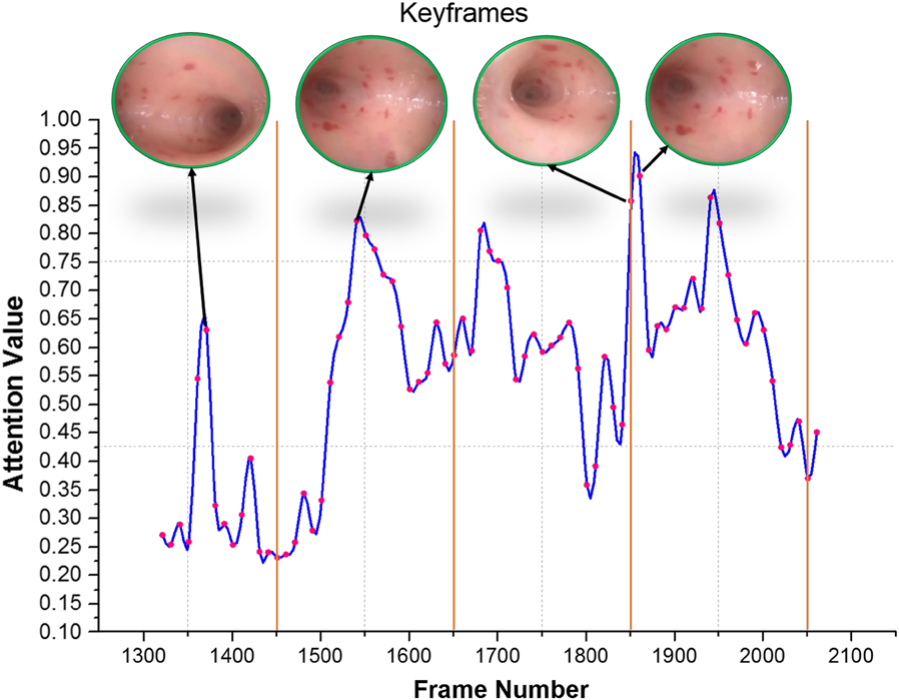
Mechanism of keyframes extraction from a sequence of diagnostic hysteroscopy frames

After computing the numerous saliencies for each frame, it is important to combine them to generate a fused saliency map for keyframes extraction. There are several ways to fuse the different saliencies such as linear fusion, linear weighted fusion, max fusion, and non-linear weighted fusion (Ejaz et al. 2013). For ease of understanding, we have used the weighted linear fusion for combining the different saliencies. To this end, the score of each saliency is normalized in the range 0–1. Then the mean of non-zero gray-levels is determined as the saliency score for each feature. The normalized values are then fused to get a final aggregated saliency score for each DH frame. Based on the fused saliency scores, an attention curve is generated, which is then used for keyframes extraction. An illustration of keyframes extraction using attention curve is given in Fig. 2.

After calculating the attention curve, the user/gynecologist is asked to specify the number of keyframes “NKF” for a given DH video. Accordingly, the video is divided into “NKF” number of shots. Within each shot, the frame with highest saliency score is determined as keyframe. By only changing the value of “NKF”, a different set of keyframes can be extracted, enabling gynecologists to analyze the DH video at different summarization levels.

## Experimental results and discussion

This sub-section illustrates the performance evaluation of various saliency detection models and general-purpose VS methods for DH videos abstraction. Experiments were performed on a set of DH videos according to Ejaz et al. (2013), each of 2–3 min duration having frame rate of 30 frames/s. MATLAB R2015a was used for conducting the experiments and running the simulation. To obtain the ground truth, gynecologists were asked to select a number of diagnostically important frames from the mentioned DH videos. In the current study, a total of five saliency detection models and two general-purpose VS schemes were considered for evaluation, in terms of keyframes extraction, F-measure (Ejaz et al. 2013), and accuracy for DH videos. These models include motion saliency model (Mehmood et al. 2015), multi-scale contrast map (Mehmood et al. 2014), texture saliency (Ejaz et al. 2013), curvature map (Mehmood et al. 2013), and SIM saliency (Bruce and Tsotsos 2005). For keyframes selection, the mean of attention values for each saliency detection scheme was considered as attention curve threshold. The frames with attention values greater than the attention curve threshold are considered as keyframes while the remaining frames are selected as non-keyframes. The frames extracted by these methods were then compared with the ground truth to find the accuracy and F-measure for each VS scheme.

**Fig. 3:**
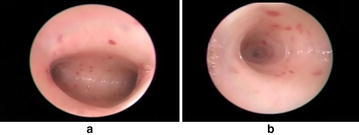
**a** The most frequently selected keyframe, **b** and least recurring keyframe

**Fig. 4:**
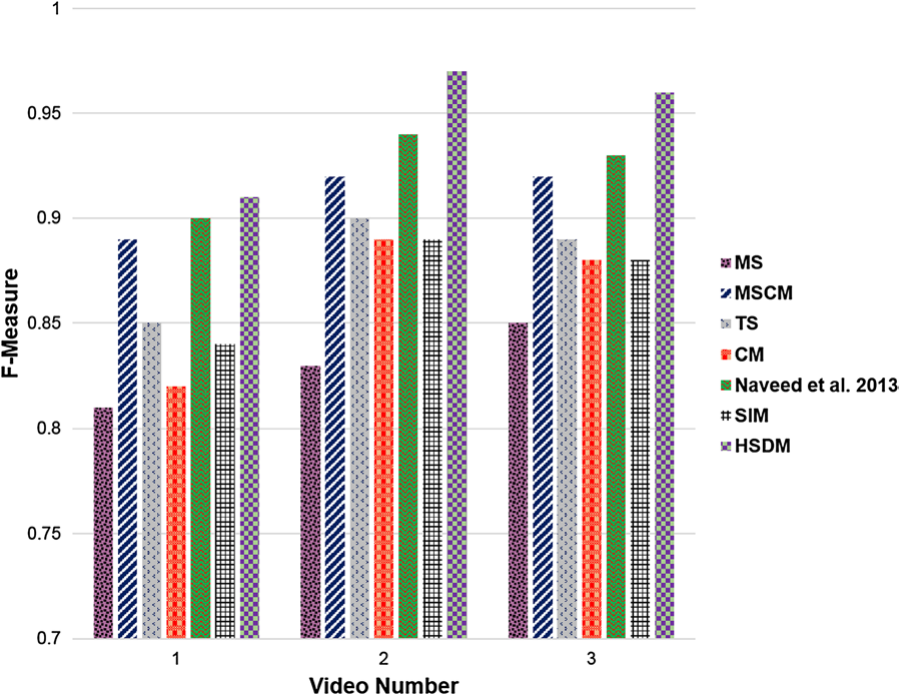
F-measure based performance evaluation of numerous saliency detection models for summarization of DH videos

Table 1 illustrates the comparative results of numerous general-purpose and domain-specific saliency detection models for summarization of DH videos. From the results, it can be seen that the performance of SIM and TS is same. Motion saliency reports 30 % accuracy, indicating worse results in this experiment. The best performance of 70 % accuracy is achieved by a hybrid visual saliency model (HSDM), consisting of motion, contrast, texture, and curvature saliencies. The most frequent and least recurring keyframes from Table 1 are shown in Fig. 3. The F-measure based performance evaluation given in Fig. 4 also verifies the fact that HSDM is comparatively more suitable for keyframes extraction from DH videos.

**Table 1:** Performance evaluation of numerous saliency detection models for a sample hysteroscopy video

Serial no.	Saliency detection model	Total keyframes	Extracted keyframes	Attention curve threshold	Accuracy (%)	Extracted frame numbers
1	MS	10	3	0.024	30	1584, 2231, 2977
2	MSCM	10	6	0.015	60	1584, 2070, 2289, 2323, 2668, 2977
3	TS	10	5	0.872	50	2070, 2264, 2289, 2323, 2977
4	CM	10	4	0.226	40	2150, 2386, 2668, 2977
5	MS + MSCM + TS	10	6	0.571	60	1584, 2070, 2231, 2323, 2386, 2668
6	HSDM	10	7	0.847	*70*	1584, 2231, 2264, 2323, 2386, 2668, 2977
7	SIM	10	5	0.984	50	1584, 2070, 2231, 2289, 2977

The score in italic font represents the best accuracy among the given methods

**Fig. 5:**
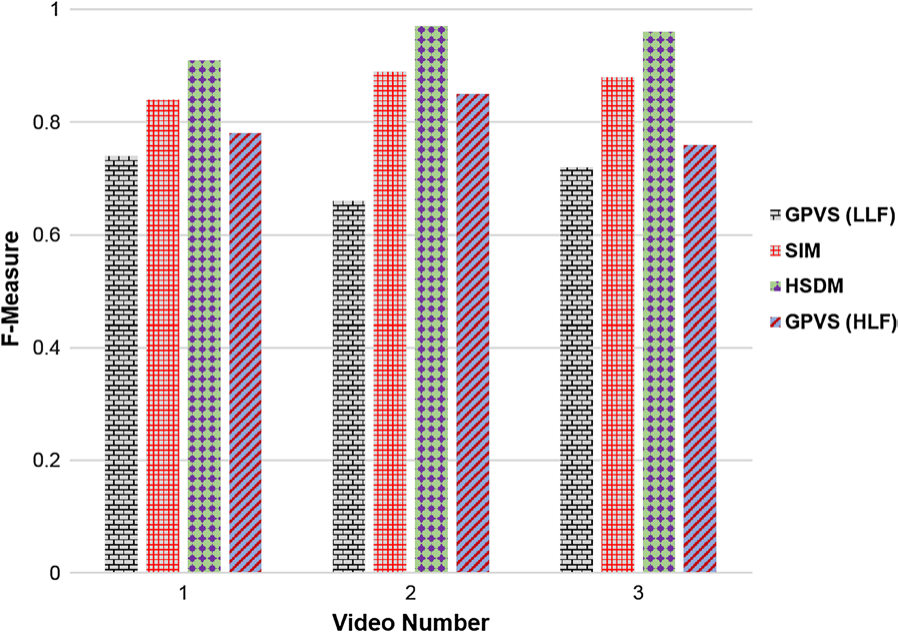
Performance evaluation of different summarization methods based on F-measure for DH videos

Table 2 presents a comparison of general VS methods, general-purpose saliency detection method, and domain-specific saliency detection schemes. The former category includes two VS methods, utilizing low and high-level features, respectively. The second scheme is a general-purpose saliency detection method which is used here for keyframes extraction from DH videos. The latter category illustrates a hybrid saliency detection framework, specific for DH videos. From the experiments, it can be noted that the suggested HSDM produces promising results by giving an accuracy of 70 %, hence dominating other related VS approaches. The same fact is also verified using F-measure based performance evaluation as given in Fig. 5.

**Table 2:** Comparison of general video summarization methods, general-purpose and domain-specific saliency detection based summarization schemes for keyframes extraction from a sample hysteroscopy video

Serial. no	Method name	Category of video summarization	Total keyframes	Feature extraction model	Total number of frames	Accuracy (%)
1	Ejaz et al. (2012)	General-purpose VS	10	Low-level features	3529	20
2	Ejaz and Baik (2012)	General-purpose VS	10	High-level features	3529	40
3	SIM (Bruce and Tsotsos 2005)	General-purpose VS	10	General-purpose saliency detection	3529	50
4	Hybrid saliency detection model	Domain-specific VS	10	Domain-specific saliency detection	3529	*70*

The score in italic font represents the best accuracy among the given methods

**Fig. 6:**
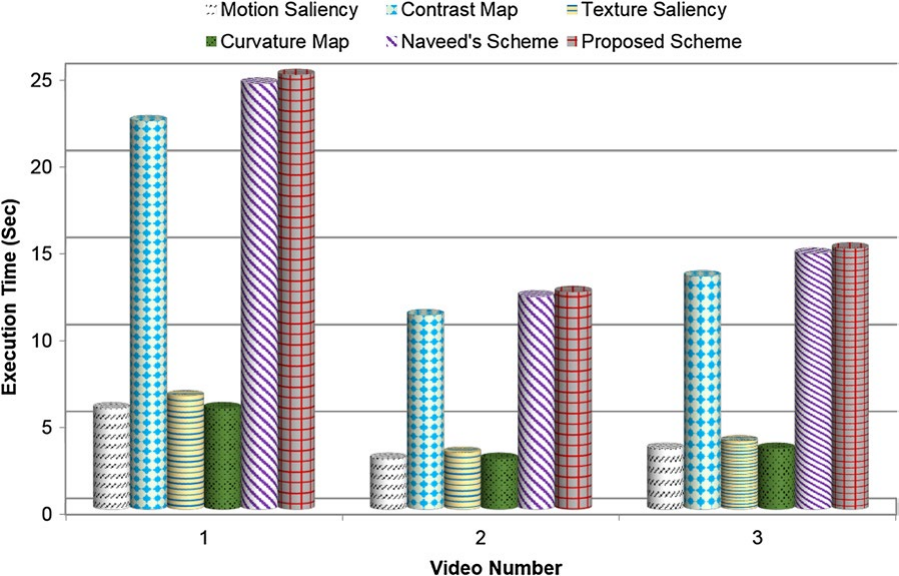
Execution time analysis for various saliency detection models

Figure 6 highlights the computational complexity of numerous saliency detection models in terms of execution time for keyframes extraction based on a set of DH videos. The graph indicates that the running time of motion, texture, and curvature saliency is almost same. Multi-scale contrast map is computationally expensive compared to the former saliencies. The running time of the suggested HSDM is slightly greater than Ejaz et al.’s scheme (2013) but it provides higher accuracy and F-measure compared to other general-purpose and domain specific VS methods.

## Conclusion

During the process of diagnostic hysteroscopy, several hysteroscopic sessions are conducted for a single patient per day. Due to the large number of patients and their multiple hysteroscopic sessions, an enormous amount of hysteroscopic videos are collected. However, a limited number of frames are required for actual diagnosis process, whose manual extraction by gynecologists is comparatively difficult and time consuming due to large-sized hysteroscopic videos. To facilitate the gynecologists in browsing for desired diagnostically important contents, video summarization schemes are used. In this work, we have conducted a comprehensive study of numerous generic and domain-specific video summarization schemes for hysteroscopic videos. Further, we have investigated the performance of various visual attention models combined with domain knowledge for summarization of DH videos. Our findings based on numerous experiments are reported as follows:

1. The general-purpose video summarization schemes are less suitable for hysteroscopic videos due to their significant similarity in color and texture, and absence of shot boundaries.
2. Among the evaluated visual saliency models, a hybrid saliency detection model comprising of motion, texture, multi-scale contrast, and curvature is found as the best combination of visual saliencies for hysteroscopic video abstraction, considering its accuracy and extracted keyframes.

In future, we have intension to focus on minimizing the computational complexity of the system by extracting light-weight features from DH videos. Another possible future direction is to combine data hiding [watermarking (Liu et al. 2015; Liu et al. 2016), image and video steganography (Mstafa and Elleithy 2015; Muhammad et al. 2015; Lin et al. 2015)] with the video summarization frameworks by embedding the patient and gynecologists data in DH videos/keyframes, resulting in secure and privacy-preserving VS framework as presented in (Muhammad et al. 2015) for secure visual contents retrieval from personalized repositories and other mobile healthcare applications (Lv et al. 2016). Furthermore, we are also planning to explore deep learning and incorporate GPUs based processing (Mei and Tian 2016; Mei 2014) for efficient keyframes extraction, their indexing and retrieval (Rho et al. 2008; Rho et al. 2011; Rho and Hwang 2006).

## Footnotes

1 Discrete Cosine Transform.
